# Prognostic value of plasma phenylalanine and gut microbiota-derived metabolite phenylacetylglutamine in coronary in-stent restenosis

**DOI:** 10.3389/fcvm.2022.944155

**Published:** 2022-08-30

**Authors:** Yuan Fu, Yixing Yang, Chen Fang, Xinming Liu, Ying Dong, Li Xu, Mulei Chen, Kun Zuo, Lefeng Wang

**Affiliations:** Heart Center, Beijing Key Laboratory of Hypertension, Beijing Chaoyang Hospital, Capital Medical University, Beijing, China

**Keywords:** in-stent restenosis (ISR), coronary artery disease (CAD), gut microbiota (GM), metabolome, phenylacetylglutamine (PAGln)

## Abstract

**Objective:**

This study was designed to explore the predictive value of plasma phenylalanine (Phe) and gut microbiota-derived metabolite phenylacetylglutamine (PAGln) in coronary in-stent restenosis (ISR).

**Methods:**

Patients with coronary ISR, in-stent hyperplasia (ISH), and in-stent patency (ISP) were retrospectively enrolled in this study. Multivariable logistic regression analyses were used to identify independent risk factors of ISR. The predictive value of plasma Phe and PAGln levels was evaluated by receiver operating characteristic (ROC) curve analysis. The areas under the ROC curve (AUCs) were compared using the Z-test. The correlation between PAGln and clinical characteristics were examined using Spearman's correlation analysis.

**Results:**

Seventy-two patients (mean age, 64.74 ± 9.47 years) were divided into three groups according to coronary stent patency: ISR (*n* = 28), ISH (*n* = 11), and ISP (*n* = 33) groups. The plasma levels of Phe and PAGln were significantly higher in the ISR group than in the ISP group. PAGln was positively associated with the erythrocyte sedimentation rate, homocysteine, SYNTAX score, triglyceride to high-density lipoprotein ratio, Phe, and microbiota-related intermediate metabolite phenylacetic acid (PA). In the ISR group, with the aggravation of restenosis, PAGln levels were also elevated. In multivariate regression analyses, Phe, PAGln and SYNTAX score were independent predictors of coronary ISR (all *P* < 0.05). In the ROC curve analyses, both Phe [AUC = 0.732; 95% confidence interval (CI), 0.606–0.858; *P* = 0.002] and PAGln (AUC = 0.861; 95% CI, 0.766–0.957; *P* < 0.001) had good discrimination performance in predicting coronary ISR, and the predictive power of PAGln was significantly better (*P* = 0.031).

**Conclusion:**

Plasma Phe and PAGln are valuable indices for predicting coronary ISR, and gut microbes may be a promising intervention target to prevent ISR progression.

## Introduction

Percutaneous coronary intervention (PCI) is the primary treatment for coronary artery disease (CAD) nowadays (1, 2). Despite the development of drug-eluting stent (DES) technologies and generations, the incidence of in-stent restenosis (ISR) remains relatively unchanged (3). ISR occurs in approximately 10% of patients after second-generation DES implantation, and the incidence of target lesion revascularization because of ISR is ~20% in 10 years (4–6). ISR is characterized by progressive luminal narrowing within the stent, which is a result of PCI-induced mechanical injury to the arterial wall of the target segment (3, 7). Aggressive neointimal hyperplasia (NIH) and late-stage neoatherosclerosis are recognized etiopathogeneses of DES-ISR (8). Although some ISR cases are clinically silent, up to 20% of cases may present with angina pectoris, and 5–10% of cases can present with acute myocardial infarction (AMI) (9, 10). Treating ISR with PCI is challenging and is associated with a higher rate of recurrent restenosis; the prognosis of patients with ISR is also worse than that of patients with *de novo* CAD (9). Given the high prevalence and poor clinical outcomes of ISR, identifying reliable predictors of coronary ISR and finding methods to prevent and treat ISR with high efficacy are important.

**Figure 1:**
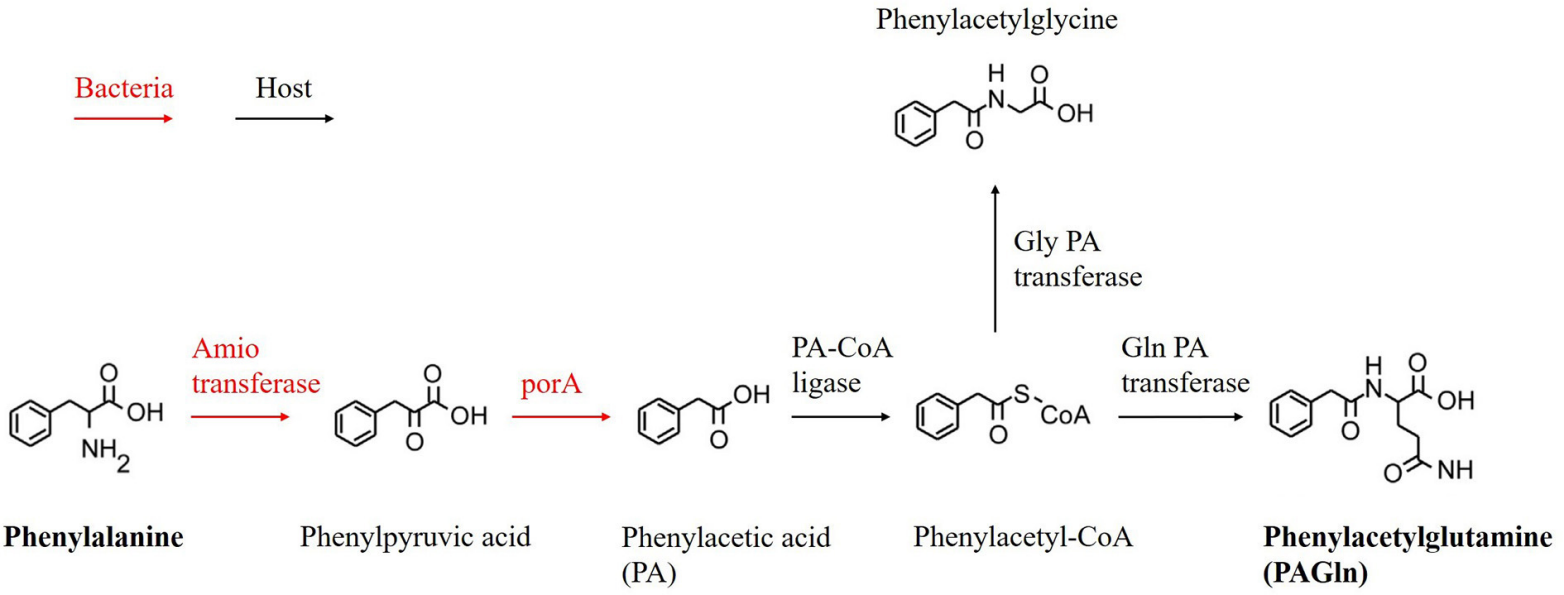
Proposed metabolic pathway for production of PAGln.

There is growing evidence that gut microbiota (GM) and its derived metabolites contribute to the pathological processes of various diseases, such as CAD, atrial fibrillation (AF), and obstructive sleep apnea (11–13). Recently, phenylacetylglutamine (PAGln), a metabolite generated by the gut microbial metabolism of phenylalanine (Phe), has been proven to be associated with enhanced platelet responsiveness and thrombosis potential (14). Studies also confirmed that circulating PAGln levels are related to coronary atherosclerotic severity and an increase in the risk of future CAD and overall mortality in patients with chronic kidney disease (CKD) (15–17). Our previous study revealed the dysbiosis of GM and PAGln in patients with CAD with in-stent stenosis (18); however, the association between substances in the entire metabolic pathway (from Phe to PAGln) (Figure 1) and coronary ISR has not been investigated.

Therefore, this study was designed to evaluate the plasma levels of Phe and its downstream metabolites and their potential relationships with coronary ISR.

## Methods

### Study population

Seventy-two consecutive patients with CAD and a previous PCI history for at least 1 year, hospitalized for rechecking coronary angiography (CAG) at the Beijing Chaoyang Hospital between June 01, 2021 and March 01, 2022, were retrospectively enrolled in this study. All patients had been successfully implanted with at least one second-generation DES before. The exclusion criteria were as follows: (1) patients with AMI or those who underwent emergency CAG; (2) patients with a history of coronary artery bypass grafting (CABG); (3) patients with a stenting vessel reference diameter of <2.5 mm; (4) patients with severe renal insufficiency or CKD; (5) patients who have used probiotics or antibiotics in the past 1 month; and (6) patients with infectious diseases, autoimmune diseases, malignant tumors, and hematological system diseases. All participants received routine secondary preventive treatment for CAD according to the modern guidelines (1, 19).

This study was approved by the Ethics Committee of Beijing Chaoyang Hospital and was conducted following the ethical standards of the 1964 Declaration of Helsinki and its later amendments. Written informed consent was obtained from all participants.

### Definition and classification of ISR

A stenosis of ≥50% of the lumen diameter within an implanted coronary stent or up to 5 mm from the stent edges is defined as angiographic ISR (3). The lumen diameter was determined by two experienced interventional cardiologists with independent double-blind visual observation, and the degree of restenosis was calculated using a general formula (20).

ISR was classified into four types: type I, focal ISR (lesions ≤10 mm in length), and the lesions can be located at the edges of the stent, within the stent, or between the neighboring stents; type II, diffuse ISR (lesions >10 mm in length), and the lesions are limited within the stent; type III, diffuse and proliferative ISR (lesions >10 mm in length), and the lesions extend beyond the stent edges; and type IV, totally occlusive ISR with thrombolysis in myocardial infarction (TIMI) grade 0 flow (3, 9).

In-stent patency (ISP) was defined as a <10% narrowing of the lumen diameter within a stent or up to 5 mm from the stent margins, and the stenosis degree of in-stent hyperplasia (ISH) was between ISR and ISP (21).

### Baseline characteristics and blood sample collection

The demographic and clinical characteristics, including age, gender, medical history, smoking and medication status, blood pressure, and body mass index (BMI), were recorded from the electronic medical records of the participants. Peripheral venous blood samples were collected on the first day after the patients' admission, at 6:00–7:00 am after overnight fasting. All blood samples were measured using a Dimension RxLMax™ automated analyzer, and biochemical variables were analyzed using a Hitachi 7,600 automatic analyzer.

After successfully puncturing the radial or femoral artery and inserting the introducer sheath, arterial blood samples (10 mL) were collected from the sheath before CAG. Then, plasma samples were isolated from arterial blood samples by centrifugation at 3,000 revolutions per min (rpm), 4°C for 10 min and stored at −80°C immediately until further analysis.

### Plasma PAGln quantification

Ultra-performance liquid chromatography coupled with triple-quadrupole tandem mass spectrometer (UPLC-QQQ-MS) technology was used for the quantitative analysis of the substances presented in Figure 1. The methyl alcohol and acetonitrile used for UPLC-QQQ-MS were purchased from Sigma-Aldrich Co., Ltd. (Missouri, USA), and the methanoic acid was purchased from Aladdin Bio-Chem Technology Co., Ltd. (Shanghai, China). All plasma samples were slowly thawed on ice, and ~100 μL of each sample was vortexed with 300-μL methyl alcohol for 30 s at 4°C. Then, the mixture was incubated at −40°C for 1 h and centrifuged at 12,000 rpm at 4°C for 15 min. The supernatant was taken, and the remaining mixture was incubated at −40°C for 1 h and centrifuged at 12,000 rpm at 4°C for 15 min again. Approximately 200 μL of the supernatant was then transferred to an injection vial for the next analysis. For UPLC, the column was an Acquity UPLC HSS T3 (1.8 μm, 2.1 mm × 100 mm), and the temperature was 40°C. The flow rate was 0.30 mL/min. The following were the mobile phases: A: water (0.1% formic acid water) and B: acetonitrile; the injection volume was 10 μL. An AB SCIEX 5500 QQQ-MS was used, and the parameters were as follows: ion source, electron spray ionization; curtain gas, 35 arbitrary units (arb) gas flow rate; collision gas, 9 arb; ion spray voltage, 4,500 V; ion source temperature, 450°C; ion source gas 1, 55 arb; and ion source gas 2, 55 arb. Multiple-reaction monitoring was then performed for quantitative substance analysis: based on the aforementioned chromatographic and MS conditions, the prepared standard solution of six substances (purchased from Yuanye Bio-Technology Co., Ltd., Shanghai, China) was added to the vial. The Rt of each substance is presented in Supplementary Table 1. The concentration of metabolites was calculated using MultiQuant Software.

### Statistical analysis

Stata (version 16.0; Stata Corporation, USA) and SPSS (version 22; IBM Corp., Armonk, NY, USA,) were used for all statistical analyses. The normal distribution of continuous variables was tested using the Kolmogorov–Smirnov test. Normally distributed data were expressed as means ± standard deviations, and abnormally distributed data were presented as medians with interquartile ranges. Student's *t-*test and the Mann–Whitney U-test were used to analyze differences in continuous variables between two groups. Categorical variables were expressed as percentages and analyzed using Pearson's chi-squared test. Spearman's correlation analysis was performed to examine the correlation between different variables. Independent risk factors of ISR were identified using multivariable logistic regression analyses. The discrimination performance of Phe and PAGln in predicting ISR was evaluated using receiver operating characteristic (ROC) curves and areas under the ROC curve (AUCs). The Youden index was calculated as follows: sensitivity + specificity – 1 (22). A Z-test was performed to compare the predictive ability (AUCs) of different variables. Two-tailed *P* < 0.05 was used to indicate statistical significance.

## Results

### Baseline characteristics of the study population

Seventy-two consecutive patients with CAD (63.9% male) after coronary DES implantation for at least 1 year were finally enrolled in this study. According to the patency of the in-stent lumen area, the patients were divided into three groups: ISR (*n* = 28; 38.89%), ISH (*n* = 11; 15.28%), and ISP (*n* = 33; 45.83%) groups. In the ISR group, 10 cases (35.71%) were ISR type I, one case (3.57%) was ISR type II, eight cases (28.57%) were ISR type III, and nine cases (32.14%) were ISR type IV. The mean age of the study population was 64.74 ± 9.47 years. The baseline clinical characteristics of the participants are presented in Table 1. The SYNTAX score was significantly higher in the ISR group than in the ISP group (20.02 ± 5.7 vs. 11.09 ± 5.6; *P* < 0.001). No significant difference in other variables was observed between the ISR and ISP groups or between the ISH and ISP groups (all *P* > 0.05).

**Table 1:** Baseline characteristics of the study population.

**Variables**	**ISR (*n* = 28)**	**ISH (*n* = 11)**	**ISP (*n* = 33)**	***P*-value 1 (ISR vs. ISP)**	***P*-value 2 (ISH vs. ISP)**
Age, years	65.32 ± 8.06	66.64 ± 7.87	63.61 ± 11.05	0.498	0.406
Male, *n*(%)	22 (78.6)	5 (45.5)	19 (57.6)	0.082	0.484
Time of stent implantation, years	7.25 (3, 9.25)	6.75 (2.5, 8.5)	6.25 (3.5, 8)	0.415	0.627
HT, *n* (%)	18 (64.3)	10 (90.9)	25 (75.8)	0.328	0.281
DM, *n* (%)	10 (55.6)	4 (36.4)	12 (36.4)	0.958	0.998
History of MI, *n* (%)	14 (50)	4 (36.4)	12 (36.4)	0.283	0.999
History of stroke, *n* (%)	5 (17.9)	1 (9.1)	9 (27.3)	0.384	0.213
Current smoker, *n* (%)	10 (55.6)	4 (36.4)	13 (39.4)	0.768	0.858
BMI, kg/m^2^	26.22 ± 3.36	26.19 ± 4.79	26.61 ± 3.21	0.646	0.743
Heart rate, bpm	71.7 ± 8.9	67.36 ± 7.3	68.94 ± 7.7	0.094	0.555
SBP, mmHg	132.29 ± 15.91	126.55 ± 11.63	133.48 ± 19.41	0.795	0.271
DBP, mmHg	75.61 ± 11.58	77.91 ± 10.13	77 ± 12.41	0.654	0.342
CCB, *n* (%)	3 (10.7)	4 (36.4)	9 (27.3)	0.105	0.567
β-RB, *n* (%)	18 (64.3)	8 (72.7)	16 (48.5)	0.216	0.162
ACEI/ARB, *n* (%)	9 (32.1)	6 (54.5)	17 (51.5)	0.127	0.862
HbA1c, (%)	6.1 (5.8,6.75)	6.3 (6, 7.3)	6.1 (5.6, 6.85)	0.376	0.143
hs-CRP, mg/L	1 (0.3, 2.4)	0.5 (0.2, 1.2)	0.8 (0.35, 1)	0.169	0.654
ESR, mm/h	6.5 (4,13.25)	8 (2, 10)	5 (2, 9)	0.195	0.588
BNP (pg/ml)	34.5 (22.25, 96)	61 (34, 78)	52 (36, 75)	0.409	0.871
WBC, *10^9^/L	6.2 ± 1.64	6.15 ± 1.63	6 ± 1.8	0.641	0.802
Hb, g/L	135.32 ± 16.49	125.64 ± 12.92	129.79 ± 16.39	0.195	0.450
Platelet, *10^9^/L	209.18 ± 41.31	190.64 ± 38.21	216.27 ± 45.69	0.610	0.229
D-dimer, mg/L FEU	0.35 (0.21, 0.45)	0.2 (0.17, 0.3)	0.3 (0.22, 0.83)	0.876	0.058
CK-MB, ng/ml	0.59 ± 0.71	0.89 ± 0.55	0.66 ± 0.61	0.658	0.271
CTnI, ng/ml	0 (0, 0.02)	0 (0, 0)	0 (0, 0)	0.197	0.237
TC, mmol/L	3.43 ± 1.11	3.27 ± 0.66	3.28 ± 0.75	0.514	0.962
HDL, mmol/L	1.05 ± 0.38	0.97 ± 0.24	1.16 ± 0.44	0.316	0.171
LDL, mmol/L	2.12 ± 1.06	1.78 ± 0.47	1.94 ± 0.53	0.405	0.393
TG, mmol/L	1.33 ± 0.49	1.34 ± 0.73	1.45 ± 0.91	0.525	0.720
TG-to-HDL ratio	1.22 (0.97, 1.72)	1.44 (0.74, 2.11)	1.1 (0.83, 1.35)	0.183	0.323
LP(a), mg/dl	15.4 (7.6, 31.08)	14.1 (7.4, 57.4)	18.4 (8.15, 24.25)	0.914	0.915
SCR, umol/L	73.35 ± 13.83	69.41 ± 10.85	67.9 ± 13.83	0.134	0.744
eGFR, ml/min/1.73 m^2^	102.57 ± 22.36	97.84 ± 15.97	108.22 ± 27.37	0.392	0.136
SUA, umol/L	344.79 ± 66.21	351.73 ± 93.49	368.88 ± 84.48	0.217	0.573
HCY, umol/L	14.88 ± 5.6	15.14 ± 4.49	12.96 ± 2.82	0.22	0.26
LAD, mm	36.5 ± 4.8	35.91 ± 3.42	38.12 ± 5.01	0.204	0.182
LVEDd, mm	47.93 ± 4.7	46.73 ± 5.75	48.09 ± 4.15	0.887	0.241
LVESd, mm	30.89 ± 5.47	27.55 ± 3.3	29.82 ± 5.4	0.444	0.197
LVEF, (%)	64.79 ± 8.74	67.09 ± 2.63	65.7 ± 6.43	0.641	0.49
SYNTAX score	20.02 ± 5.7	13.91 ± 6.14	11.09 ± 5.6	***<*** **0.001**	0.165

Data are number (%), mean (SD), or median (IQR).

ISR, in-stent restenosis; ISH, in-stent hyperplasia; ISP, in-stent patency; HT, hypertension; DM, diabetes mellitus; MI, myocardial infarction; BMI, body mass Index; SBP, systolic blood pressure; DBP, diastolic blood pressure; CCB, calcium channel blocker; β-RB, β-receptor blocker; ACEI, angiotensin-converting enzyme inhibitor; ARB, agiotensin receptor blocker; HbA1c, glycosylated hemoglobin, type A1C; hs-CRP, high-sensitivity C-reactive protein; ESR, erythrocyte sedimentation rate; BNP, B-type natriuretic peptide; WBC, white blood cell; Hb, hemoglobin; CK-MB, creatine kinase MB; CTnI, cardiac troponin I; TC, total cholesterol; HDL, high- density lipoprotein cholesterol; LDL, low-density lipoprotein cholesterol; TG, triglyceride; LP(a), lipoprotein(a); SCR, serum creatinine; eGFR, estimated glomerular filtration rate; SUA, serum uric acid; HCY, homocysteine; LAD, left atrial diameter; LVEDd, left ventricular end-diastolic dimension; LVESd, left ventricular end- systolic dimension; LVEF, Left ventricular ejection fraction.

The bold values indicate the *P* value <0.05.

### Plasma Phe and PAGln levels in different groups

Six substances in the entire metabolic pathway (Figure 1) were quantitatively analyzed (Table 2). However, two of them (i.e., phenylpyruvic acid and phenylacetyl-CoA) could not be quantified because the levels of these two substances were below the lower limit of the measure value. The levels of Phe were significantly higher in the ISR (1,535.9 ± 287.32 vs. 1,316.8 ± 250.97; *P* = 0.02) and ISH (1,699.6 ± 324.2 vs. 1,316.8 ± 250.97; *P* < 0.001) groups than in the ISP group. The level of PAGln was significantly higher in the ISR group than in the ISP group (801.12 ± 291.1 vs. 367.18 ± 271.02; *P* < 0.001); however, no statistically significant difference in PAGln levels was observed between the ISH and ISP groups (526.75 ± 256.38 vs. 367.18 ± 271.02; *P* = 0.094). The plasma levels of PAGln tended to elevate from ISR type I to ISR type IV (Table 3), and the level of PAGln was significantly higher in ISR type IV than in ISR type I (906.83 ± 300 vs. 652.54 ± 174.78; *P* = 0.045).

**Table 2:** Levels of plasma metabolites.

**Variables**	**ISR (*n* = 28)**	**ISH (*n* = 11)**	**ISP (*n* = 33)**	***P*-value 1 (ISR vs. ISP)**	***P*-value 2 (ISH vs. ISP)**
Phenylalanine, μg/mL	1,535.9 ± 287.32	1,699.6 ± 324.2	1,316.8 ± 250.97	**0.02**	**<0.001**
PA, ng/mL	72.62 (35.56, 90.12)	73.39 (56.95, 117.57)	64.4 (40.13, 115.16)	0.977	0.391
Phenylacetylglycine, ng/mL	2.63 (2.45, 4.21)	2.73 (2.45, 3.08)	2.62 (2.4, 3.24)	0.981	0.81
PAGln, ng/mL	801.12 ± 291.1	526.75 ± 256.38	367.18 ± 271.02	**<0.001**	0.094

Data are mean (SD), or median (IQR).

ISR, in-stent restenosis; ISH, in-stent hyperplasia; ISP, in-stent patency; PA, phenylacetic acid; PAGln, phenylacetylglutamine.

The bold values indicate the *P* value <0.05.

**Table 3:** Plasma phenylalanine and PAGln levels in ISR group.

**Variables**	**ISR type I (*n* = 10)**	**ISR type II (*n* = 1)**	**ISR type III (*n* = 8)**	**ISR type IV (*n* = 9)**	**ISP (*n* = 33)**	***P*-value 1**	***P*-value 2**	***P*-value 3**	***P*-value 4**
Phenylalanine, μg/mL	1,543.5 ± 212.02	1,133.4	1,502.6 ± 252.41	1,601.8 ± 382.06	1316.8 ± 250.97	**0.013**	0.477	0.068	**0.01**
PAGln, ng/mL	652.54 ± 174.78	843.7	862.61 ± 366.38	906.83 ± 300	367.18 ± 271.02	**0.003**	0.093	**<0.001**	**<0.001**

Data are mean (SD). P-value 1: ISR type I vs. ISP; P-value 2: ISR type II vs. ISP; P-value 3: ISR type III vs. ISP; P-value 4: ISR type IV vs. ISP.

PAGln, phenylacetylglutamine; ISR, in-stent restenosis; ISP, in-stent patency.

The bold values indicate the *P* value <0.05.

### Phe and PAGln were independent risk factors of ISR

In multivariate logistic regression analyses, Phe [odds ratio (OR) = 1.001; 95% confidence interval (CI), 1.000–1.002; *P* = 0.01) and PAGln (OR = 1.008; 95% CI, 1.003–1.014; *P* = 0.003] were two independent predictors of ISR, after adjusting for age, gender, BMI, diabetes mellitus (DM), left ventricular ejection fraction (LVEF) and SYNTAX score (Table 4). In other two regression models, Phe (OR= 1.000; 95% CI, 1.000–1.000; *P* = 0.012) and PAGln (OR = 1.008; 95% CI, 1.003–1.013; *P* = 0.003) also remained as independent predictors of ISR, after adjusting for age, gender, BMI, LVEF, glycosylated hemoglobin, type A1c (HbA1c) level and SYNTAX score (Table 4).

**Table 4:** Multiple logistic regression analyses for independent risk factors of ISR.

	**Estimated β**	**OR (95% CI)**	***P*-value**
Model 1			
SYNTAX score	0.331	1.393 (1.153–1.683)	0.001
Phenylalanine	0.001	1.001 (1.000–1.002)	0.01
Model 2			
SYNTAX score	0.317	1.372 (1.143–1.648)	0.001
Phenylalanine	0	1.000 (1.000–1.000)	0.012
Model 3			
SYNTAX score	0.44	1.553 (1.144–2.11)	0.005
PAGln	0.008	1.008 (1.003–1.014)	0.003
Model 4			
SYNTAX score	0.434	1.544 (1.142–2.088)	0.005
PAGln	0.008	1.008 (1.003–1.013)	0.003

Variables included in model 1 are age, gender, BMI, DM, LVEF, SYNTAX score and Phenylalanine.

Variables included in model 2 are age, gender, BMI, HbA1c level, LVEF, SYNTAX score and Phenylalanine.

Variables included in model 3 are age, gender, BMI, DM, LVEF, SYNTAX score and PAGln. Variables included in model 4 are age, gender, BMI, HbA1c level, LVEF, SYNTAX score and PAGln.

BMI, body mass Index; DM, diabetes mellitus; LVEF, left ventricular ejection fraction; HbA1c, glycosylated hemoglobin, type A1C; PAGln, phenylacetylglutamine.

### Correlation between PAGln and clinical characteristics

**Figure 2:**
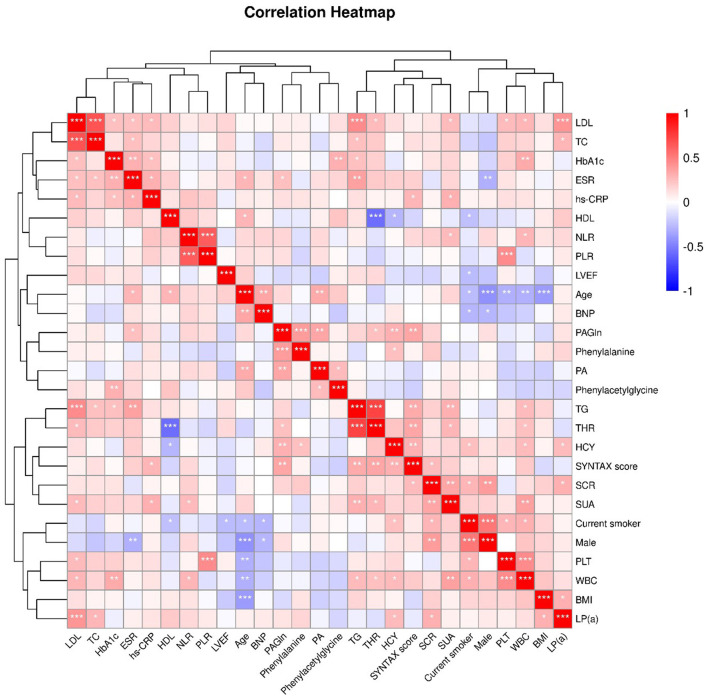
Heatmap showing the correlation between clinical characteristics. The correlation coefficient (Spearman's correlation analysis) is expressed in different colors, Red, positive correlation, Blue, negative correlation. *, *P* <0.05; **, *P* < 0.01; ***, *P* < 0.001. LDL, low-density lipoprotein cholesterol; TC, total cholesterol; HbA1c, glycosylated hemoglobin, type A1C; ESR, erythrocyte sedimentation rate; hs-CRP, high-sensitivity C-reactive protein; HDL, high- density lipoprotein cholesterol; NLR, neutrophil-lymphocyte ratio; PLR, platelet- lymphocyte ratio; LVEF, Left ventricular ejection fraction; BNP, B-type natriuretic peptide;PAGln, phenylacetylglutamine; PA, phenylacetic acid; TG, triglyceride; THR, TG-HDL ratio; HCY, homocysteine; SCR, serum creatinine; SUA, serum uric acid; PLT, platelet; WBC, white blood cell; BMI, body mass Index; LP(a), lipoprotein(a).

Spearman's correlation analysis was performed, and the results are presented in Figure 2. As a product of the metabolic pathway, PAGln was positively correlated with Phe [correlation coefficient (CC) = 0.394; *P* < 0.001] and the intermediate metabolite phenylacetic acid (PA) (CC = 0.343; P = 0.003) (Figure 1). Moreover, PAGln was also positively correlated with several clinical characteristics, including erythrocyte sedimentation rate (ESR) (CC = 0.244; P = 0.039), homocysteine (HCY) (CC = 0.303; *P* = 0.009), SYNTAX score (CC = 0.328; *P* = 0.005), and triglyceride to high-density lipoprotein ratio (THR) (CC = 0.301; *P* = 0.047).

### ROC curve analyses

**Figure 3:**
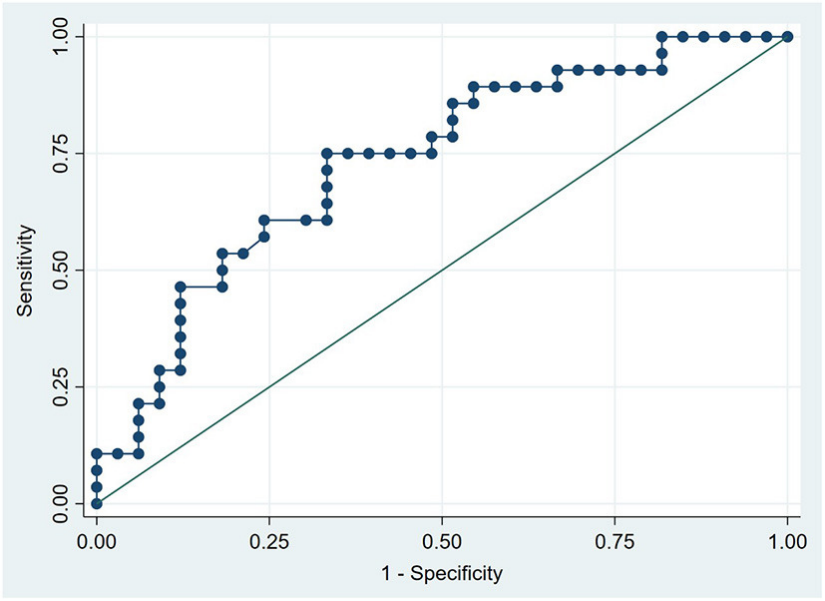
ROC curve analysis for the plasma phenylalanine level in predicting ISR. The AUC was 0.732 (95% CI: 0.606–0.858, *P* = 0.002).

**Figure 4:**
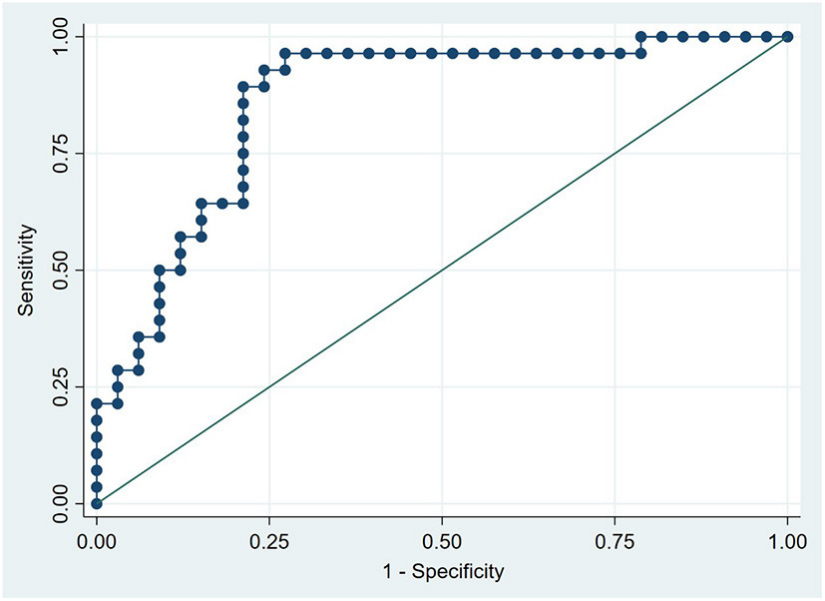
ROC curve analysis for the plasma PAGln level in predicting ISR. The AUC was 0.861 (95% CI 0.766–0.957, *P* < 0.001).

**Figure 5:**
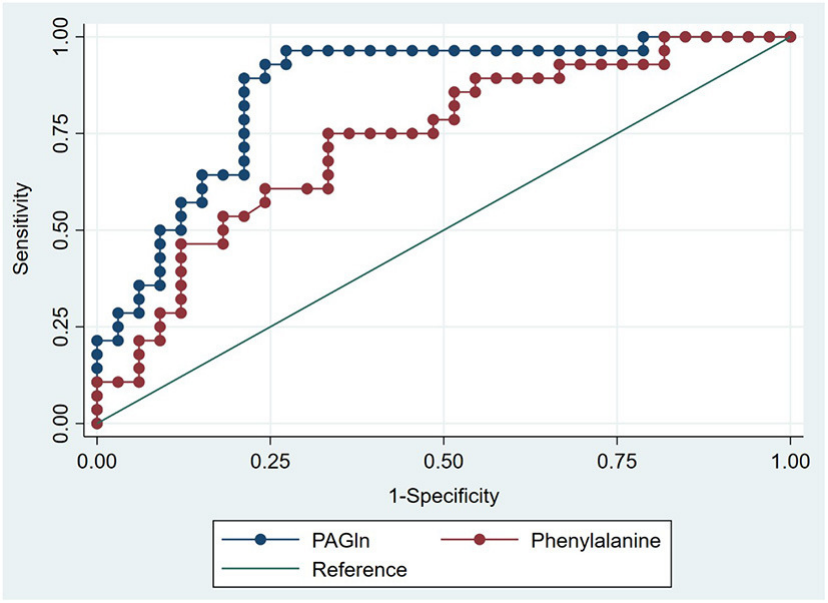
The comparison of AUCs using Z test. The AUC of plasma PAGln level in predicting ISR was significantly higher than that of plasma phenylalanine level (*P* = 0.031).

ROC curve analyses were performed, and the AUC of plasma Phe and PAGln levels in predicting ISR was 0.732 (95% CI, 0.606–0.858; P = 0.002) (Figure 3) and 0.861 (95% CI, 0.766–0.957; *P* < 0.001) (Figure 4), respectively. The maximum value of the Youden index was a criterion for best cutoff value selection (23), and the best cutoff values of Phe and PAGln for predicting ISR were 1,370.48 μg/mL (sensitivity: 75%, specificity: 66.7%) and 423.63 ng/mL (sensitivity: 96.4%, specificity: 72.7%), respectively. Both Phe and PAGln had good discriminatory power in predicting ISR, and after the Z-test, the predictive value of PAGln was significantly better (*P* = 0.031) (Figure 5).

## Discussion

In this study, we demonstrated that the plasma levels of Phe and its downstream GM-related metabolite PAGln were independently associated with coronary ISR. Both indices showed good discrimination performance in predicting ISR, and the predictive value of PAGln was significantly better.

PCI has become a major therapeutic method for CAD over the past few decades, but coronary ISR remains an unsolved clinical problem (3, 8). Despite the continuous improvements in stent design, polymers, and antiproliferative drugs with modern DESs, approximately 10% of the patients treated with PCI are likely to develop target lesion failure because of ISR (3, 7, 8). ISR is a result of PCI procedure-induced mechanical injury to the blood vessel wall, and the main etiopathogenesis of ISR is intimal hyperplasia and late neoatherosclerosis (7, 8). ISR affects the quality of life of patients with PCI and is independently associated with poor prognosis; meanwhile, treating ISR is difficult and challenging (9, 10). Given the rapidly increasing application of PCI worldwide and the high prevalence and adverse outcomes of ISR, it is crucial to further understand the pathophysiological causes of ISR and identify risk factors for developing ISR.

Recently, dysbiosis of GM and altered circulating GM-related metabolites have been identified as important contributing factors for the pathological process of several cardiovascular diseases (24, 25). For instance, trimethylamine N-oxide (TMAO), a gut microbial metabolite, has been demonstrated to be associated with extremely late stent thrombosis, coronary plaque rupture, platelet hyperreactivity, and future CAD risk (26–29). Short-chain fatty acids (SCFAs), another kind of significant GM-derived metabolites, have also been proved to play important roles in the pathological process of hypertensive cardiovascular damage, AF, and AMI (30–32).

PAGln, a metabolite formed from the conjugation of glutamine and phenylacetate, which is almost exclusively generated from bacterial microbial Phe metabolism, has become a very recent research hotspot (14). A study by Nemet et al. has shown that plasma PAGln is associated with the incidence of cardiovascular disease and major adverse cardiovascular events (MACEs), after adjusting for traditional cardiac risk factors (14). The pathophysiological link between PAGln and MACEs was partly because of enhanced platelet responsiveness and thrombosis potential (14, 33). A study by Liu et al. confirmed the independent association between plasma PAGln levels and the degree of coronary atherosclerotic severity (16). Another study suggested that higher plasma levels of PAGln are associated with the moderate-severe overall white matter hyperintensity in patients with acute ischemic stroke (34). However, the association between ISR and plasma PAGln levels has rarely been examined.

Our previous study preliminarily discussed the close correlation between GM dysbiosis, altered plasma PAGln levels, and ISR in patients with CAD (18). Based on the results of this previous study, we conducted this study to further examine the association between PAGln and coronary ISR: we comprehensively investigated the relationship between ISR and all substances in the metabolic pathway (Figure 1). Four substances (i.e., Phe, PA, PAGln, and phenylacetylglycine) were finally quantified, and the ISR and ISH groups had higher plasma levels of Phe than the ISP group. ISH can be regarded as the prophase of ISR, and the plasma levels of PAGln were rising from ISP to ISH to ISR (progression of stenosis degree). Significantly elevated plasma PAGln levels were observed in the ISR group compared with those in the ISP group; however, no significant elevation was observed in the ISH group. The levels of another two substances, PA and phenylacetylglycine, did not differ among the three groups. This may be because these two substances are involved in multiple metabolic pathways, which thus influence their plasma levels. Moreover, from ISR type I to ISR type IV (progression of stenosis degree), the plasma levels of PAGln were also rising. The plasma levels of PAGln were significantly higher in ISR type IV patients than in ISR type I patients (906.83 ± 300 vs. 652.54 ± 174.78; *P* = 0.045). Given these results, we believe that circulating PAGln could promote the progressive luminal narrowing within the stent, from patency to hyperplasia and finally to stenosis in patients with CAD after PCI treatment. The relatively small sample size might account for the statistically insignificant differences in PAGln levels between some groups (e.g., ISP and ISH).

Phe is an essential amino acid enriched in high-protein diets and can be metabolized by GM to generate phenylpyruvic acid and then PA (14). After the absorption of PA into the portal system, the host liver can metabolize PA into PAGln; therefore, limiting the intake of Phe or intervening the GM may be reasonable methods for reducing plasma PAGln levels, thus preventing the progression of ISR.

The underlying mechanism, through which plasma PAGln may participate in the pathogenesis of ISR remains unclear. A study by Menni et al. found that PAGln is strongly related to the gene expression level of the cell death activator CIDE, which is a gene associated with insulin resistance in adipose tissue (35). Insulin resistance affects vascular endothelial function and neointimal tissue proliferation after coronary stent implantation, in both patients with and without diabetes, and may thus promote the progression of ISR (36, 37). Moreover, gut microbial PAGln synthesis-related enzyme genes abundant in ISR patients were positively correlated with systemic inflammation level, and inflammation plays an important role in the development of ISR (18). Additionally, PAGln enhances platelet activation by triggering adrenergic receptor signaling (14). Activated platelets can release various biomolecules that promote the dedifferentiation of vascular smooth muscle cells and may ultimately result in NIH and ISR (38, 39). However, the exact mechanism underlying the close association between circulating PAGln level and ISR should be examined in future cellular and animal experiments.

No statistical differences in the baseline clinical characteristics, expect for SYNTAX score, were observed among the three groups (i.e., ISR, ISH, and ISP). In multivariate logistic regression analyses after adjusting for baseline clinical characteristics (i.e., age, gender, BMI, DM, HbA1c and LVEF) and SYNTAX score (a characteristic with a statistically significant difference between the ISR and ISP groups), both PAGln and Phe remained as independent risk factors for ISR. DM is a well-known risk factor for the development of coronary ISR, and good glycaemic control to obtain optimal HbA1c level is crucial to reduce the incidence of ISR after PCI (40, 41). However, both DM and HbA1c were not remained as independent risk factors of ISR in the present study after multivariate logistic regression analyses. This is because the proportion of DM patients and HbA1c levels did not statistically differ between ISR and ISP groups. Furthermore, PAGln was independently correlated with ESR, HCY, SYNTAX score, and THR, all of which were reported to be associated with coronary ISR in previous studies (5, 42–44). In the ROC curve analysis, an AUC of 1.0 indicates perfect discriminatory performance, while an AUC < 0.5 represents the absence of predictive ability (22). The discrimination performance of PAGln in predicting ISR was excellent (AUC = 0.861; 95% CI, 0.766–0.957; *P* < 0.001) and significantly better than that of Phe (AUC = 0.732; 95% CI, 0.606–0.858; *P* = 0.002). The best cutoff point of PAGln to predict ISR was 423.63 ng/mL, with a sensitivity of 96.4% and specificity of 72.7%. The aforementioned results indicated that plasma PAGln is a valid biomarker in predicting coronary ISR with high accuracy; however, prospective large-scale studies are still needed to verify the predictive value of PAGln.

### Limitations

This study has some limitations. First, the sample size was relatively small, and the potential cause–effect relationship could not be estimated. Second, the causal link between PAGln and ISR could not be determined. Third, we did not collect fecal samples; the GM composition and function may have not been represented. Fourth, ISR is a chronic, time-dependent process; only one measurement of the plasma level of PAGln may not represent the entire pathophysiological course. Finally, operator-related and mechanical factors also affect the process of ISR, and this study was insufficient to analyze these aspects of effects.

## Conclusion

Elevated plasma levels of Phe and PAGln were independent predictors of ISR in patients with CAD with a PCI history. Both biomarkers showed good discriminatory power in predicting ISR, and the predictive value of PAGln was significantly better. This study provides a promising target for preventing and treating coronary ISR from the perspective of dietary and GM regulation. Prospective studies validating the discriminatory ability of PAGln to predict ISR and studies focusing on the causal link between PAGln and ISR are needed in the future.

## Data availability statement

The raw data supporting the conclusions of this article will be made available by the authors, without undue reservation.

## Ethics statement

The studies involving human participants were reviewed and approved by the Ethics Committee of Beijing Chaoyang Hospital. The patients/participants provided their written informed consent to participate in this study.

## Author contributions

YF and YY: study design, data analysis, and manuscript writing. CF, XL, and YD: collected and analyzed part of the data. LX and MC: provided critical review. KZ and LW: provided technical support and commented on the manuscript drafts. All authors read and approved the manuscript.

## Funding

This work was funded by Beijing Municipal Administration of Hospitals (XXZ0607).

## Conflict of interest

The authors declare that the research was conducted in the absence of any commercial or financial relationships that could be construed as a potential conflict of interest.

## Publisher's note

All claims expressed in this article are solely those of the authors and do not necessarily represent those of their affiliated organizations, or those of the publisher, the editors and the reviewers. Any product that may be evaluated in this article, or claim that may be made by its manufacturer, is not guaranteed or endorsed by the publisher.

## Abbreviations

Phe: phenylalanine
PAGln: phenylacetylglutamine
ISR: in-stent restenosis
ISH: in-stent hyperplasia
ISP: in-stent patency
ROC: receiver operating characteristic
AUCs: areas under the ROC curve
PA: phenylacetic acid
PCI: percutaneous coronary intervention
CAD: coronary artery disease
DES: drug-eluting stent
NIH: neointimal hyperplasia
AMI: acute myocardial infarction
GM: gut microbiota
AF: atrial fibrillation
CKD: chronic kidney disease
CAG: coronary angiography
CABG: coronary artery bypass grafting
BMI: body mass index
rpm: revolutions per minute
UPLC-QQQ-MS: ultra-performance liquid chromatography coupled with triple-quadrupole tandem mass spectrometer
arb: arbitrary units
OR: odds ratio
CI: confidence interval
DM: diabetes mellitus
LVEF: left ventricular ejection fraction
HbA1c, glycosylated hemoglobin: type A1c
CC: correlation coefficient
ESR: erythrocyte sedimentation rate
HCY: homocysteine
TMAO: trimethylamine N-oxide
SCFAs: Short-chain fatty acids
MACEs: major adverse cardiovascular events.
