# Supplementary material for: Prognostic value of plasma phenylalanine and gut microbiota-derived metabolite phenylacetylglutamine in coronary in-stent restenosis
Source: Front Cardiovasc Med. 2022 Aug 30;9:944155. doi: 10.3389/fcvm.2022.944155 (PMC9468445; doi:10.3389/fcvm.2022.944155)
Supplement: Supplementary file 1 [file Data_Sheet_1.docx]

Supplementary table 1. Multiple-reaction monitoring acquisition parameters

| Q1 Mass (Da) | Q3 Mass (Da) | Substances | Rt (minutes) |  |
| --- | --- | --- | --- | --- |
| 166.1 | 103 | Phenylalanine | 2.99 |  |
| 163.8 | 92.1 | Phenylpyruvic acid | 4.02 |  |
| 135.4 | 91 | Phenylacetic acid (PA) | 4.01 |  |
| 886.1 | 379.2 | Phenylacetyl-CoA | 4.21 |  |
| 886.1 | 428.3 | Phenylacetyl-CoA | 4.21 |  |
| 192 | 74 | Phenylacetylglycine | 3.31 |  |
| 192 | 91 | Phenylacetylglycine | 3.31 |  |
| 263.9 | 127 | Phenylacetylglutamine (PAGln) | 3.03 |  |
| 263.9 | 145 | Phenylacetylglutamine (PAGln) | 3.03 |  |
